# Comparative effectiveness and durability of COVID‐19 vaccination against death and severe disease in an ongoing nationwide mass vaccination campaign

**DOI:** 10.1002/jmv.27934

**Published:** 2022-06-23

**Authors:** Theodore Lytras, Flora Kontopidou, Angeliki Lambrou, Sotirios Tsiodras

**Affiliations:** ^1^ Department of Medicine, School of Medicine European University Cyprus Nicosia Cyprus; ^2^ National Public Health Organization Athens Greece; ^3^ 4th Department of Internal Medicine, Attikon University Hospital, Medical School National and Kapodistrian University of Athens Athens Greece

**Keywords:** cohort studies, COVID‐19, COVID‐19 vaccines, immunogenicity, pandemic, SARS‐CoV‐2, vaccination, vaccine effectiveness

## Abstract

As national coronavirus disease 2019 (COVID‐19) mass vaccination campaigns are rolled out, monitoring real‐world Vaccine Effectiveness (VE) and its durability is essential. We aimed to estimate COVID‐19 VE against severe disease and death in the Greek population, for all vaccines currently in use. Nationwide active surveillance and vaccination registry data during January–December 2021 were used to estimate VE via quasi‐Poisson regression, adjusted for age and calendar time. Interaction terms were included to assess VE by age group, against the “delta” severe acute respiratory syndrome coronavirus 2 variant and waning of VE over time. Two doses of BNT162b2, mRNA‐1273, or ChAdOx1 nCov‐19 vaccines offered very high (>90%) VE against both intubation and death across all age groups, similar against both “delta” and previous variants, with one‐dose Ad26.COV2.S slightly lower. VE waned over time but remained >80% at 6 months, and three doses increased VE again to near 100%. Vaccination prevented an estimated 19 691 COVID‐19 deaths (95% confidence interval: 18 890–20 788) over the study period. All approved vaccines offer strong and also durable protection against COVID‐19 severe disease and death. Every effort should be made to vaccinate the population with at least two doses, to reduce the mortality and morbidity impact of the pandemic.

## 1 INTRODUCTION

The efficacy of coronavirus disease 2019 (COVID‐19) vaccines in preventing symptomatic infection has been documented in multiple clinical trials^1^, ^2^, ^3^, ^4^ and observational studies.^5^, ^6^ However, as the severe acute respiratory syndrome coronavirus 2 (SARS‐CoV‐2) transitions towards endemicity,^7^ preventing severe COVID‐19 through vaccination becomes a strategic priority to reduce overall disease burden and mortality and safeguard healthcare services. Therefore monitoring COVID‐19 Vaccine Effectiveness (VE) against severe disease and death in a real‐life setting is essential, to quantify the benefits of vaccination, generate confidence in the public and promote uptake. Long‐term observational VE studies can further provide much‐needed answers about the durability of protection, and its extent against different SARS‐CoV‐2 variants.

Since December 2020 Greece has rolled out a centralized nationwide mass vaccination campaign (Table S1 for a detailed timeline), offering four COVID‐19 vaccines to its entire resident population free‐of‐charge; two messenger RNA (mRNA) vaccines, BNT162b2 (Pfizer‐BioNTech) and mRNA‐1273 (Moderna), and two adenoviral vector vaccines, ChAdOx1 nCoV‐19 (AstraZeneca) and Ad26.COV2.S (Janssen). This offers the opportunity to study the comparative real‐life effectiveness of these vaccines in the same population and setting.

The objective of this cohort study was to estimate COVID‐19 VE against severe disease and death for all available vaccine/dose combinations, stratified by age group, in the entire Greek population. Additional objectives were to detect waning of VE over time, and to estimate VE against the “delta” SARS‐CoV‐2 variant (vs previously circulating variants).

## 2 MATERIALS AND METHODS

### 2.1 Study population and data collection

In Greece, the National Public Health Organization (EODY) is responsible for COVID‐19 surveillance, with particular emphasis on active case finding and follow‐up of all severe cases nationwide. A severe COVID‐19 case is defined as a laboratory‐confirmed case (using PCR or antigen testing) that has been intubated and/or hospitalized in intensive care or has died (regardless of setting). As the availability of intensive care beds varied during the pandemic, we focused on intubation and death as the two most unbiased and completely ascertained outcomes. COVID‐19 deaths were defined under World Health Organization guidelines as those with laboratory confirmation and clinically compatible illness without complete recovery preceding death, and with no time cut‐off following laboratory confirmation.^8^

For the study period between January 11, 2020 and December 8, 2021 we obtained anonymized surveillance data for all COVID‐19 intubations and deaths in Greece in persons aged 15 years and older (9.2 million population), including their vaccination status at the time of laboratory confirmation as reported to EODY by the National Vaccine Registry (NVR). The NVR is operated by the Hellenic Ministry of Digital Governance and registers all COVID‐19 vaccinations performed in Greece, without exception. From the NVR we additionally obtained aggregate counts of persons vaccinated with every vaccine/dose combination, for every age and every day during the study period, and further stratified by time since vaccination; this allowed accurate measurement of person‐time spent in each vaccination “state,” and thus calculation of crude and age‐adjusted rates of COVID‐19 intubations and deaths for each vaccination group, based on the dates of laboratory confirmation (rather than dates of intubation or death).

No funding was received for this study. The study was approved by the EODY board; as only anonymized data were used from which no person can be identified, no separate ethical approval was required. The board had no role in study design, analysis, interpretation of data, and decision to publish.

### 2.2 Statistical analysis

VE against COVID‐19 intubation and against COVID‐19 death was estimated using quasi‐Poisson regression as one minus the Incidence Rate Ratio (IRR), adjusted for age (in 5‐year groups) and calendar time (calendar week as categorical variable). Two models were fitted: model A, grouping all vaccines together and comparing 1‐dose, 2‐dose, and 3‐dose vaccination to the unvaccinated, and model B, assessing separately each vaccine/dose combination among those that had at least 10 events and 10 000 person‐years of associated follow‐up. Both models included additional interaction terms: (a) between vaccine and age (15–59, 60–79, and 80+ years) to detect any diminished effectiveness among the elderly; (b) between vaccine and time since 1 month after the last received dose, to assess waning of VE compared to the first month after vaccination; and (c) between vaccine and prevalence of the “delta” SARS‐CoV‐2 variant. Data on the latter were provided by the National SARS‐CoV‐2 Genomic Surveillance Network, coordinated by EODY; before ISO week 25/2021 “delta” was absent, and after week October 2021 fully dominant, while for the intermediate period (Weeks 25–30/2021) the proportion of “delta” among all randomly selected and genotyped samples was used (Table S2). In case of severe collinearity (Variance Inflation Factor over 5) or sparse data (<5 tabulated events) the respective interaction terms were dropped from model B. Also the interaction term between vaccine and time since the last dose was dropped for 3‐dose vaccination as follow‐up time was too short (Table S3). Stratified VE estimates were calculated as linear combinations of the respective model coefficients. We additionally used the fitted models to estimate the number of intubations and deaths prevented by vaccination during the study period, as the predicted number of outcome events if everyone was unvaccinated, minus observed events.^9^ All statistical analyses were performed with the R statistical environment, version 4.1.2.

## 3 RESULTS

**Figure 1:**
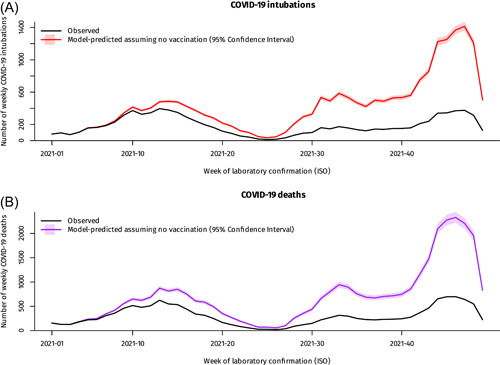
Weekly COVID‐19 deaths and intubations in Greece, January–December 2021, observed vs model‐predicted assuming no vaccination. Footnote for Figure 1: The latest weeks in the series are subject to reporting delay. COVID‐19, coronavirus disease 2019.

During the study period, a total of 14 676 605 vaccine doses were administered in Greece (11 427 784 BNT162b2, 1 161 905 mRNA‐1273, 1 505 334 ChAdOx1 nCoV‐19 and 581 582 Ad26.COV2.S) and a total of 9100 COVID‐19 intubations and 14 755 COVID‐19 deaths occurred. Event rates and follow‐up time per vaccination group are detailed in Table 1; although a substantial number of vaccinated persons died of COVID‐19, crude and age‐adjusted rates were several times lower compared to the unvaccinated group. Vaccinated individuals, especially those that received three doses, were also older on average than the unvaccinated (Table 1). Weekly rates of COVID‐19 intubation and death varied greatly over time, reflecting SARS‐CoV‐2 community prevalence and spread (Figure 1).

**Table 1:** Crude and age‐adjusted rates of COVID‐19 intubation and death by vaccination group, Greece, January 1–December 8, 2021

				Intubations			Deaths		
Vaccination group*	Person‐years of follow‐up	Median follow‐up, days (IQR)	Median age, years (IQR)**	Number of intubations	Rate per 100k person‐years	Age‐adjusted rate per 100k person‐years	Number of deaths	Rate per 100k person‐years	Age‐adjusted rate per 100k person‐years
Unvaccinated	5 138 482	N/A	45 (30–60)	7984	155.38	92.82	12 172	236.88	141.52
3‐dose (any vaccine)	106 773	15 (6–30)	75 (61–81)	24	22.48	0.28	52	48.70	0.60
2‐dose (any vaccine)	2 638 990	167 (133–191)	58 (43–72)	684	25.92	7.95	1806	68.44	21.00
1‐dose (any vaccine)	720 464	21 (20–27)	50 (35–63)	408	56.63	4.74	725	100.63	8.43
2‐dose BNT162b2	2 037 824	168 (131–194)	57 (43–74)	548	26.89	6.37	1629	79.94	18.94
2‐dose ChAdOx1 nCov‐19	341 120	166 (150–185)	61 (43–64)	101	29.61	1.17	133	38.99	1.55
1‐dose BNT162b2	331 326	20 (20–21)	52 (36–68)	272	82.09	3.16	499	150.61	5.80
2‐dose mRNA‐1273	251 492	175 (149–194)	55 (44–70)	35	13.92	0.41	42	16.70	0.49
1‐dose Ad26. COV2.S	184 317	148 (90–176)	39 (26–49)	41	22.24	0.48	110	59.68	1.28
1‐dose ChAdOx1 nCoV‐19	157 366	76 (61–79)	61 (43–64)	66	41.94	0.77	87	55.29	1.01
3‐dose BNT162b2	91 089	18 (7–35)	75 (60–82)	24	26.35	0.28	51	55.99	0.59
1‐dose mRNA‐1273	47 455	27 (27–28)	52 (40–67)	29	61.11	0.34	29	61.11	0.34

Abbreviations: COVID‐19, coronavirus disease 2019; IQR, interquartile range; mRNA, messenger RNA.

* Only combinations with at least 10 events (deaths or intubations) and 10 000 person‐years of associated follow‐up. Rates are calculated based on the dates of laboratory confirmation, with person‐years spent in each vaccination state as denominator.

** Distribution weighted by follow‐up time.

**Figure 2:**
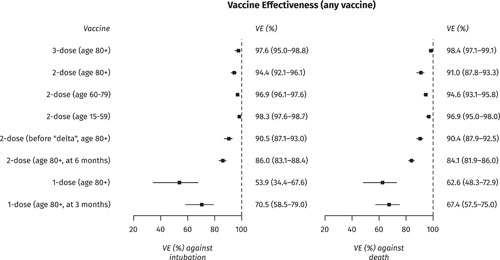
Effectiveness of 1‐, 2‐ and 3‐dose vaccination against COVID‐19 intubation and death, Greece, January–December 2021 (Model A). COVID‐19, coronavirus disease 2019; VE, Vaccine Effectiveness.

Adjusted VE estimates from models A and B are presented in Figures 2, S1, and S2. Two COVID‐19 vaccine doses offered more than 90% protection against death and intubation across all age groups, with marginally lower protection in persons 80 years or older. VE against death was similar for both “delta” and previously circulating variants, and even slightly higher against intubation from the “delta” variant (*p* < 0.001). However, 2‐dose VE waned slightly over time while still being over 80% at 6 months, and a third vaccine dose restored it close to 100% even in the oldest age group. Effectiveness for one vaccine dose was suboptimal but still sufficient to halve the rate of both death and intubation compared with the unvaccinated (Figure 2). Both the BNT162b2, mRNA‐1273, and ChAdOx1 nCoV‐19 vaccines showed similar and very high VE (>90%) in all age groups, with a slight waning over time that was marginally better with mRNA‐1273 particularly among younger adults (Figure S2). In contrast, one dose of Ad26.COV2.S offered significantly less protection against intubation or death during the first month after vaccination, similar to that of one dose of BNT162b2; over time, however, VE for Ad26.COV2.S increased and at 6 months was comparable to that of the other 2‐dose vaccines.

**Figure 3:**
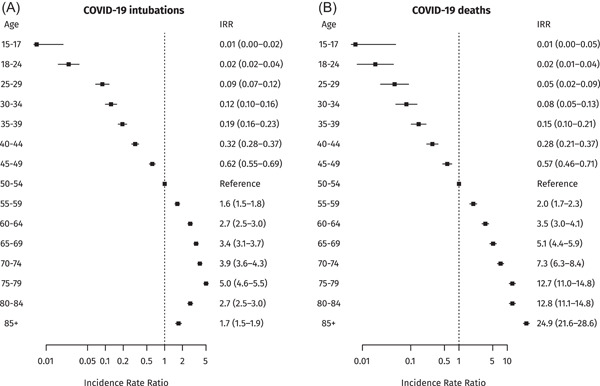
Rates of COVID‐19 intubation and death by age group, Greece, January–December 2021 (Model B). COVID‐19, coronavirus disease 2019.

Given the above associations from Model B, if vaccination was not available the pandemic would have had a much more severe impact, especially during the large “delta” wave of winter 2021 (Figure 1); over the study period vaccination prevented an estimated 19 691 COVID‐19 deaths (95% confidence interval [CI]: 18 890–20 788) and 6674 COVID‐19 intubations (95% CI: 6251–7241). Finally, there was a very steep age gradient in the rate of COVID‐19 death, with more than a thousand‐fold difference between the younger and older age groups; a similar gradient was observed for intubations but the rate was lower in people older than 80 years, suggesting clinicians may avoid invasive mechanical ventilation in severely ill elderly patients (Figure 3).^10^

## 4 DISCUSSION

Our study provides important real‐world validation for the remarkable effectiveness and durability of COVID‐19 vaccination against severe disease or death. VE was similar and very high (>90%) across all age groups, despite the absolute benefit being higher for older people due to their much higher susceptibility. Furthermore, effectiveness was similar against the “delta” variant of SARS‐CoV‐2 compared to “alpha” and older variants, as seen in other populations.^11^, ^12^ The findings confirm that vaccination is by far the most important public health tool to blunt the mortality and morbidity impact of the COVID‐19 pandemic, prevent overloading of healthcare services and save lives.^13^

With the large size and long follow‐up of our study population, we were able to demonstrate a small, yet statistically significant, waning of effectiveness against severe disease and death. Even in the oldest age group though, VE 6 months after two doses of mRNA or ChAdOx1 nCoV‐19 vaccine remained higher than 80%, a very substantial level of protection, which is restored by a third dose to levels even higher than the initial 2‐dose vaccination. Our findings are very similar to a recent study from England using the test‐negative design.^14^ Notably though, the difference in VE between 2‐dose and 3‐dose vaccination is much smaller than between 1‐dose and 2‐dose, or 1‐dose and being unvaccinated; therefore for public health authorities, closing the existing COVID‐19 vaccination gaps is an even more urgent priority than offering third doses to the population.^15^ While the benefit from a third dose needs to be balanced against the ethical and utilitarian need for global vaccine equity,^16^ an additional challenge is presented by the “omicron” variant given the substantial effectiveness of third doses in preventing “omicron” symptomatic infection.^17^ Therefore to meet global demand, vaccine manufacturers have to urgently scale up production.

The four examined vaccines showed only marginal differences in effectiveness. Interestingly, VE for 1‐dose Ad26.COV2.S is consistent with its postvaccination antibody response, which is initially lower but more stable over time.^18^ The similar VE for all vaccines is important, as there are no head‐to‐head randomized trials, and provides valuable reassurance to the public who might ask which vaccine is best. It also shifts considerations about specific vaccine recommendations away from effectiveness and primarily towards availability, logistics, and safety profile issues.

Strengths of our study include the large sample size, long follow‐up, and practically complete ascertainment of both vaccination and severe outcomes across the entire population of Greece, minimizing selection bias or bias due to health‐seeking behavior. Detailed information on age enabled tight adjustment for confounding; given the very large effect of age on the risk of severe COVID‐19 disease and death, residual confounding by age is an important concern and may lead to VE underestimation. Adjustments for calendar time and time since vaccination allowed accounting for waning effectiveness and the variable SARS‐CoV‐2 spread in the community, which was not always possible in other studies.

However, there are certain limitations. We did not examine laboratory‐confirmed COVID‐19 cases to estimate VE against infection, as these are highly dependent on health‐seeking behavior and testing patterns, thus susceptible to bias. We did not have information on comorbidities or socioeconomic status; these are possible confounders since they are associated with both vaccination and risk of COVID‐19,^19^ although comorbidities are also largely associated with age, for which adjustment was made. Finally, we did not have information on previous SARS‐CoV‐2 infection; since infected persons were recommended to receive a single dose of COVID‐19 vaccine, which creates strong “hybrid” immunity,^20^ this might have led to some overestimation of 1‐dose VE in particular. Furthermore, as infections accumulate over time and induce some immunity among the unvaccinated, VE would be expected to attenuate; the fact that it remains very high throughout the study period is further testament to the remarkable effectiveness of COVID‐19 vaccination.

In conclusion, our study provides valuable documentation of the very high and durable effectiveness of COVID‐19 vaccination in preventing severe disease and death in all age groups, both against “delta” and older SARS‐CoV‐2 variants. The findings support the efforts to promote vaccination uptake, thereby reducing the impact of the COVID‐19 pandemic. Formal studies to monitor the effectiveness of COVID‐19 vaccination are essential, as simple comparisons of counts or rates in surveillance data will underestimate effectiveness primarily due to confounding by age. Finally, as the “omicron” variant and potential newer variants emerge, our study provides a blueprint for long‐term monitoring of vaccine effectiveness and its durability over the next phase of the pandemic.

## AUTHOR CONTRIBUTIONS

*Original idea*: Theodore Lytras and Sotirios Tsiodras. *Data collection*: Flora Kontopidou and Angeliki Lambrou. *Data analysis*: Theodore Lytras. *Data interpretation*: All authors. *First draft of the manuscript*: Theodore Lytras. *Revision of the manuscript for important intellectual content*: All authors.

## CONFLICTS OF INTEREST

The authors declare no conflicts of interest.

## ETHICS STATEMENT

The study was approved by the EODY board; as only anonymized data were used from which no person can be identified, no separate ethics approval was required.

## Supporting information

Effectiveness of 1‐, 2‐ and 3‐dose vaccination against COVID‐19 death and intubation, Greece, January‐December 2021 (full results – all vaccines grouped). ***Footnote for Supplementary Figure 1***: All results pertain to the “delta” variant, unless otherwise indicated.

Comparative effectiveness of BNT162b2, mRNA‐1273, ChAdOx1 nCoV‐19 and Ad26.COV2.S vaccines against COVID‐19 death and intubation, Greece, January‐December 2021 (full results – individual complete vaccinations). ***Footnote for Supplementary Figure 2***: All results pertain to the “delta” variant, unless otherwise indicated.

Supplementary information.

Supplementary information.

Supplementary information.

## Data Availability

The data that support the findings of this study are available from the Hellenic Ministry of Digital Governance and the National Public Health Organization. Restrictions apply to the availability of these data, which were used under license for this study. Data are available from the authors with the permission of the Hellenic Ministry of Digital Governance and the National Public Health Organization.
