# Supplementary figures and images for: Comparative effectiveness and durability of COVID‐19 vaccination against death and severe disease in an ongoing nationwide mass vaccination campaign
Source: J Med Virol. 2022 Jun 23;94(10):5044–50. doi: 10.1002/jmv.27934 (PMC9349766; doi:10.1002/jmv.27934)

## Vaccine Effectiveness (any vaccine)

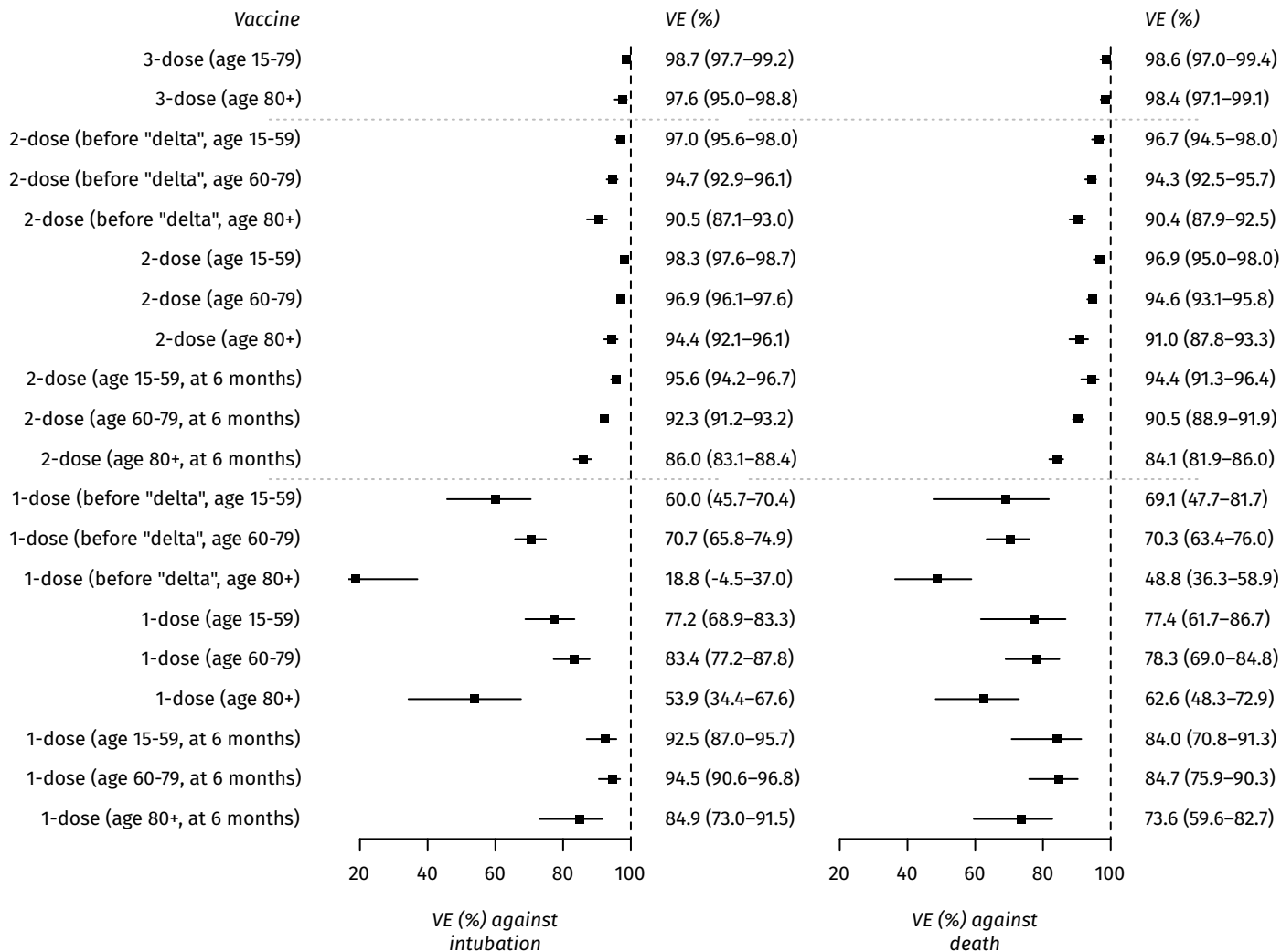

Supplement: Supplementary file 1 — Effectiveness of 1‐, 2‐ and 3‐dose vaccination against COVID‐19 death and intubation, Greece, January‐December 2021 (full results – all vaccines grouped). Footnote for Supplementary Figure 1 : All results pertain to the “delta” variant, unless otherwise indicated. [file JMV-94-5044-s001.pdf]

# Vaccine Effectiveness (comparative)

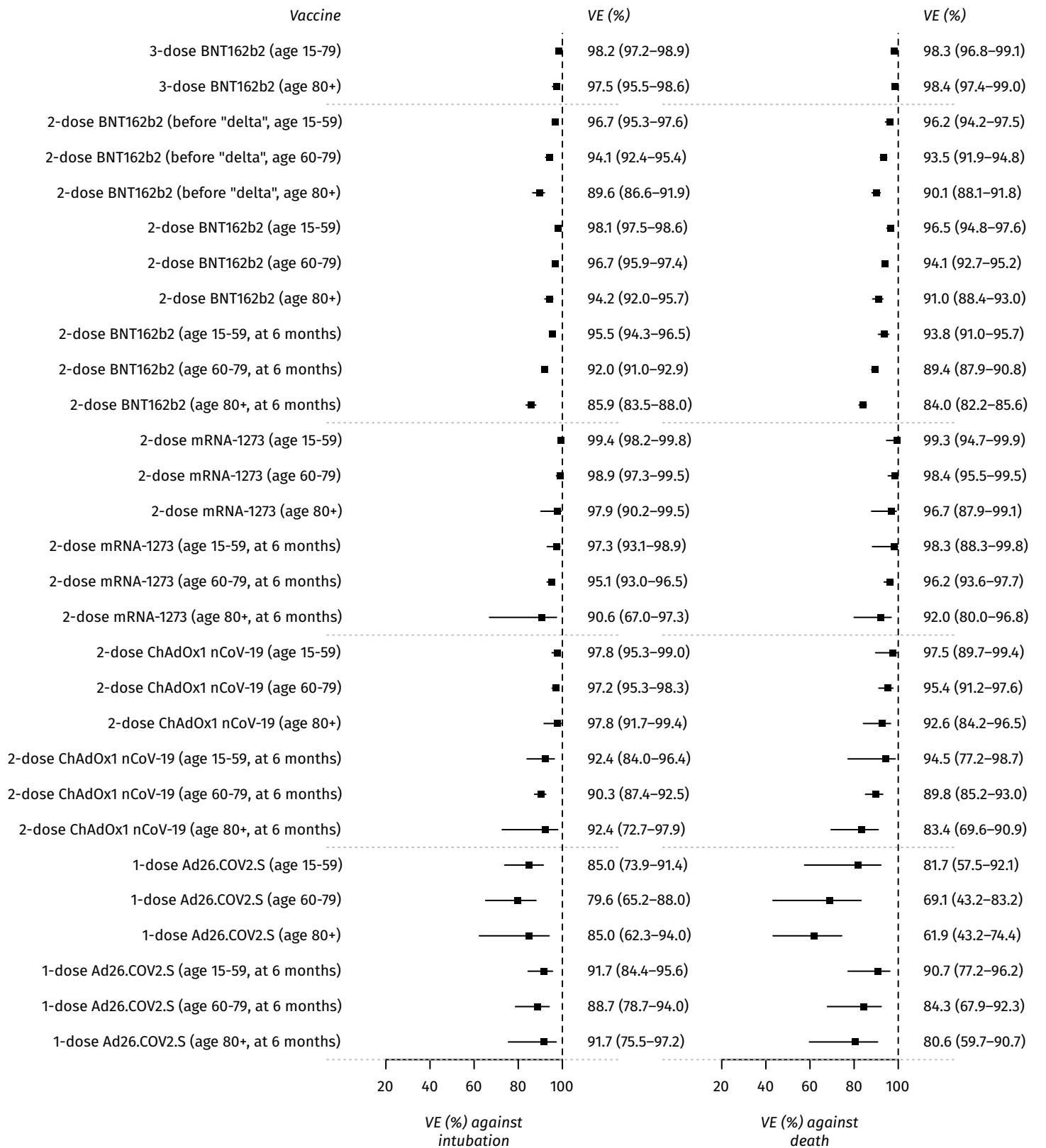

Supplement: Supplementary file 2 — Comparative effectiveness of BNT162b2, mRNA‐1273, ChAdOx1 nCoV‐19 and Ad26.COV2.S vaccines against COVID‐19 death and intubation, Greece, January‐December 2021 (full results – individual complete vaccinations). Footnote for Supplementary Figure 2 : All results pertain to the “delta” variant, unless otherwise indicated. [file JMV-94-5044-s003.pdf]
