# Supplementary material for: Comparative effectiveness and durability of COVID‐19 vaccination against death and severe disease in an ongoing nationwide mass vaccination campaign
Source: J Med Virol. 2022 Jun 23;94(10):5044–50. doi: 10.1002/jmv.27934 (PMC9349766; doi:10.1002/jmv.27934)
Supplement: Supplementary file 3 — Supplementary information. [file JMV-94-5044-s005.docx]

**Supplementary Table 1:** Timeline of vaccine rollout in Greece

| **Date** | **Rollout** |
| --- | --- |
| 20 December 2020 | Healthcare workers |
| 11 January 2021 | Ages 85+, all available vaccines |
| 22 January 2021 | Ages 80-84, all available vaccines |
| 10 February 2021 | Ages 60-64, ChAdOx1 nCoV-19 vaccine only |
| 12 February 2021 | Ages 75-79, all available vaccines |
| 26 March 2021 | Ages 70-74, all available vaccines |
| 2 April 2021 | Ages 65-69, all available vaccines |
| 10 April 2021 | Ages 60-64, all available vaccines |
| 21 April 2021 | Ages 50-54, all available vaccines |
| 24 April 2021 | Ages 55-59, all available vaccines |
| 27 April 2021 | Ages 30-39, ChAdOx1 nCoV-19 vaccine only |
| 29 April 2021 | Ages 40-44, all available vaccines |
| 1 May 2021 | Ages 45-49, all available vaccines |
| 26 May 2021 | Ages 35-39, all available vaccines |
| 29 May 2021 | Ages 30-34, all available vaccines  Ages 18+, Ad26.COV2.S vaccine only |
| 10 June 2021 | Ages 25-29, all available vaccines except ChAdOx1 nCoV-19 |
| 16 June 2021 | Ages 18-24, all available vaccines except ChAdOx1 nCoV-19 |
| 15 July 2021 | Ages 15-17, BNT162b2 vaccine only |
| 30 July 2021 | Ages 12-14, BNT162b2 vaccine only |
| 14 September 2021 | Third vaccine dose, for immunocompromised patients, BNT162b2 or mRNA-1273 vaccine only |
| 30 September 2021 | Third vaccine dose, healthcare workers and ages 60+, BNT162b2 or mRNA-1273 vaccine only |
| 10 October 2021 | Third vaccine dose, ages 50+, BNT162b2 or mRNA-1273 vaccine only |
| 5 November 2021 | Repeat dose for Ad26.COV2.S recipients, Ad26.COV2.S or BNT162b2 or mRNA-1273 vaccine |
| 20 November 2021 | Third vaccine dose, ages 18+, BNT162b2 or mRNA-1273 vaccine only |
| 10 December 2021 | Ages 5-11, pediatric BNT162b2 vaccine only |

The above timeline was determined by vaccine availability and the recommendations of the Greek National Commitee for Immunizations.
