# Supplementary material for: Comparative effectiveness and durability of COVID‐19 vaccination against death and severe disease in an ongoing nationwide mass vaccination campaign
Source: J Med Virol. 2022 Jun 23;94(10):5044–50. doi: 10.1002/jmv.27934 (PMC9349766; doi:10.1002/jmv.27934)
Supplement: Supplementary file 4 — Supplementary information. [file JMV-94-5044-s002.docx]

**Supplementary Table 2:** Proportion of the “delta” SARS-CoV-2 variant among randomly selected and genotyped SARS-CoV-2 samples, Greece, National SARS-CoV-2 Genomic Surveillance Network

| **Week(s)** | **Samples with “Delta” variant** | **Samples with other variants** | **“Delta” proportion (%)** |
| --- | --- | --- | --- |
| 20-24/2021 | 26 | 4,389 | 0.6 |
| 25/2021 | 20 | 249 | 8.0 |
| 26/2021 | 155 | 484 | 32.0 |
| 27/2021 | 741 | 1,341 | 55.3 |
| 28/2021 | 1,474 | 1,969 | 74.9 |
| 29/2021 | 1,435 | 1,630 | 88.0 |
| 30/2021 | 930 | 1,013 | 91.8 |
| 31-35/2021 | 3,795 | 3,861 | 98.3 |

Before week 25/2021, the most common circulating variants were B.1.1.7 (“Alpha”) and B.1.1.318.
