# Supplementary material for: Comparative effectiveness and durability of COVID‐19 vaccination against death and severe disease in an ongoing nationwide mass vaccination campaign
Source: J Med Virol. 2022 Jun 23;94(10):5044–50. doi: 10.1002/jmv.27934 (PMC9349766; doi:10.1002/jmv.27934)
Supplement: Supplementary file 5 — Supplementary information. [file JMV-94-5044-s004.docx]

**Supplementary Table 3:** Follow-up distribution per vaccination group (days)

|  | Quantile | | | | |
| --- | --- | --- | --- | --- | --- |
| Vaccine group | 2.5% | 25% | 50% | 75% | 97.5% |
| 1-dose BNT162b2 | 0 | 6 | 12 | 18 | 121 |
| 1-dose mRNA-1273 | 0 | 7 | 15 | 23 | 120 |
| 1-dose ChAdOx1 nCoV-19 | 1 | 18 | 37 | 56 | 135 |
| 1-dose Ad26.COV2.S | 3 | 34 | 73 | 118 | 179 |
| 2-dose BNT162b2 | 4 | 41 | 85 | 134 | 222 |
| 2-dose mRNA-1273 | 4 | 43 | 86 | 132 | 197 |
| 2-dose ChAdOx1 nCoV-19 | 4 | 41 | 83 | 126 | 180 |
| 3-dose BNT162b2 | 0 | 6 | 15 | 30 | 59 |
